# Role of p53 in Cell Death and Human Cancers

**DOI:** 10.3390/cancers3010994

**Published:** 2011-03-03

**Authors:** Toshinori Ozaki, Akira Nakagawara

**Affiliations:** 1 Laboratory of Anti-tumor Research, Chiba Cancer Center Research Institute, 666-2 Nitona, Chuoh-ku, Chiba 260-8717, Japan; E-Mail: tozaki@chiba-cc.jp; 2 Division of Biochemistry and Laboratory of Innovative Cancer Therapeutics, Chiba Cancer Center Research Institute, 666-2 Nitona, Chuoh-ku, Chiba 260-8717, Japan

**Keywords:** apoptosis, DNA damage, p53

## Abstract

p53 is a nuclear transcription factor with a pro-apoptotic function. Since over 50% of human cancers carry loss of function mutations in *p53* gene, p53 has been considered to be one of the classical type tumor suppressors. Mutant p53 acts as the dominant-negative inhibitor toward wild-type p53. Indeed, mutant p53 has an oncogenic potential. In some cases, malignant cancer cells bearing *p53* mutations display a chemo-resistant phenotype. In response to a variety of cellular stresses such as DNA damage, p53 is induced to accumulate in cell nucleus to exert its pro-apoptotic function. Activated p53 promotes cell cycle arrest to allow DNA repair and/or apoptosis to prevent the propagation of cells with serious DNA damage through the transactivation of its target genes implicated in the induction of cell cycle arrest and/or apoptosis. Thus, the DNA-binding activity of p53 is tightly linked to its tumor suppressive function. In the present review article, we describe the regulatory mechanisms of p53 and also p53-mediated therapeutic strategies to cure malignant cancers.

## 1. Introduction

Cells are constantly exposed to a variety of cellular stresses such as DNA damage. These cellular stresses finally introduce the genomic aberrations including mutation, deletion and/or translocation into the cellular genome and thereby induce the genomic instability. Accumulation of the genomic aberrations often results in the development of cancers [1]. Therefore, a proper stress response is required to maintain the genomic integrity and protect cells from malignant transformation.

p53 is a nuclear transcription factor and transactivates numerous target genes involved in the induction of cell cycle arrest and/or apoptosis [2-5]. Under normal conditions, p53 is expressed at an extremely low level, which is caused by proteasomal degradation mediated largely by RING-finger type E3 ubiquitin protein ligase MDM2 [6-8] and as a functionally latent form. Upon DNA damage, p53 is induced to accumulate in cell nucleus through post-translational modifications such as phosphorylation and acetylation. These chemical modifications convert p53 from a latent to an active form, which might be due to the dissociation of MDM2 from p53 [2-5]. Functionally active p53 transactivates an appropriate set of its target genes to induce cell cycle arrest and/or apoptosis, which is dependent on the extent and types of DNA damage [2-5,9]. p53-mediated cell cycle arrest allows cells to repair damaged DNA. When DNA repair is complete, cells re-enter the normal cell cycle. In contrast, when cells have serious DNA damage, p53 exerts its pro-apoptotic function to eliminate cells with serious DNA damage and thereby inhibit the transfer of damaged DNA to daughter cells. Thus, p53 has an ability to maintain genomic integrity.

Extensive mutation searches demonstrated that over 50% of human cancers carry the loss of function mutations in *p53* gene [10-17], suggesting that p53 is a classical Knudson-type tumor suppressor. Indeed, *p53*-deficient mice developed spontaneous cancers [18]. Among them, 95% of mutations are detectable within the genomic region encoding the DNA-binding domain of p53 and thereby mutant p53 lacks the sequence-specific transactivation ability [4]. The transactivation ability of p53 is tightly linked to its pro-apoptotic function [19,20]. Mutant p53 forms the hetero-oligomer with wild-type p53 through their intact oligomerization domains and acts as a dominant-negative inhibitor toward wild-type p53 [21,22]. In contrast to short-lived wild-type p53 (20 min), mutant p53 has a prolonged half-life (2 to 12 h) with an oncogenic potential [23-25]. Cancers bearing *p53* mutations sometimes display the chemo-resistant phenotype, indicating that p53 plays a critical role in the regulation of DNA damage response [26-28]. Thus, it is quite important to develop a novel strategy to eliminate the negative effect of mutant p53 on wild-type p53 for efficient chemotherapy.

## 2. Structural Features of p53

p53 is composed of three representative functional domains including an NH_2_-terminal acidic transactivation domain (TA: amino acid residues 1–45), a DNA-binding domain (DB: amino acid residues 102–292) and a COOH-terminal oligomerization domain (OD: amino acid residues 319–359) [2-5]. The NH_2_-terminal transactivation domain is subdivided into two independent domains such as TA 1 (amino acid residues 1-40) and TA II (amino acid residues 43–63) [29,30]. The DNA-binding domain binds to the tandem repeat of the p53-responsive element (RRRCWWGYYY: R, G/A; W, A/T; Y, C/T) separated by up to 13 bp within the promoter regions of its target genes [19]. In response to a variety of cellular stresses, such as DNA damage and energetic stress, p53 induces cell cycle arrest and/or apoptosis through the transactivation of its target genes. Functional p53 forms a homo-tetramer, which is mediated by its COOH-terminal oligomerization domain.

In addition to those representative functional domains, p53 contains several characteristic domains required for its activity. Since p53 is a transcription factor, p53 must be localized within cell nucleus. For the efficient nuclear access of p53, p53 contains three nuclear localization signals (NLSs, amino acid residues 305–322, 369–375 and 379–384), which are recognized by importin α/β complex [31]. Dysfunction of importin α resulted in the cytoplasmic retention of p53 [32]. Therefore, importin α contributes to the nuclear localization of p53. Alternatively, p53 contains a Leu-rich nuclear export signal (NES, amino acid residues 339–352) recognized by nuclear export machinery CRM1 (chromosomal region maintenance 1) [31]. CRM1 is a member of the karyo-pherin-β family of receptor proteins and has been proposed as being the key factor that mediates the nuclear export of p53 [33]. It has been shown that the tetramer formation of p53 masks NES and thereby inhibits its nuclear export [34]. In contrast, MDM2-mediated monoubiquitination at the COOH-terminal Lys residues disrupted tetramer formation of p53 and exposed NES for CRM1 binding [35]. p53 also has a Pro-rich domain (amino acid residues 63–97). This pro-rich domain has been shown to be associated with pro-apoptotic function of p53 [36,37]. Indeed, deletion of this pro-rich region led to a complete loss of pro-apoptotic activity of p53.

## 3. Mutational Inactivation of p53

Mutational inactivation is considered to be one of the most common molecular mechanisms behind the dysfunction of p53. Extensive mutation search revealed that more than half of human cancers carry loss of function mutations of p53 [16]. Among them, 95% of mutations were detectable within the genomic region (exons 5–8) encoding the DNA-binding domain [4]. The close inspection of the mutation profiles revealed that the six amino acid residues are most frequently mutated in human cancers including Arg-175, Gly-245, Arg-248, Arg-249, Arg-273 and Arg-282 [38]. These mutations found within the DNA-binding domain of p53 disrupt its proper conformation and thus the mutant p53 is defective in the sequence-specific transcriptional activation dependent on the wild-type p53-binding consensus element. Furthermore, mutant p53 displays a dominant-negative behavior toward wild-type p53 through the formation of hetero-tetramer with wild-type p53 and has oncogenic potential [21,22,25].

**Figure 1.**
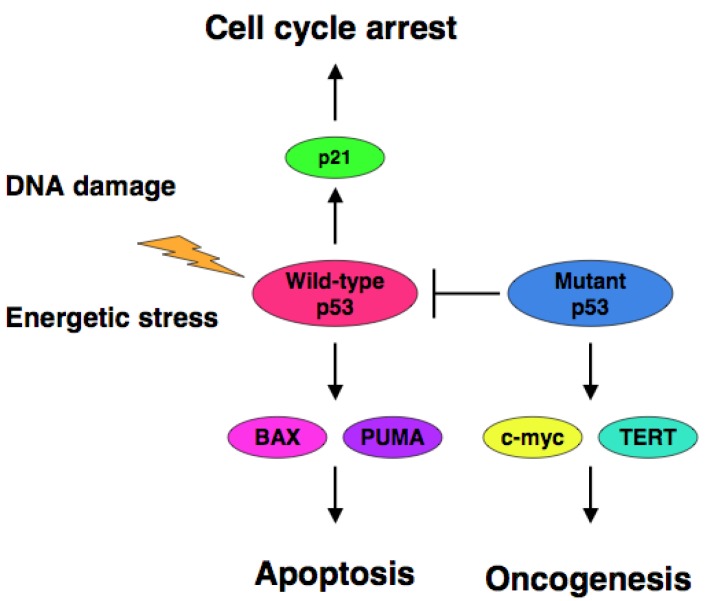
Dominant-negative effect of mutant p53 on wild-type p53. Pro-apoptotic function of p53 is significantly inhibited by certain p53 mutants which induce malignant transformation through up-regulation of c-myc and TERT.

The accumulating evidence demonstrated that certain cancer-derived mutant forms of p53 transactivate various target genes such as the multiple drug resistance gene 1 (MDR1), c-myc, proliferating cell nuclear antigen (PCNA), interleukin-6 (IL-6), insulin-like growth factor 1 (IGF-1), fibroblast growth factor (FGF) and epidermal growth factor receptor (EGFR) [39-45]. Scian *et al.* found that cancer-derived mutant p53 transactivates aspargine synthetase (ASNS) and telomerase reverse transcriptase (TERT) [46]. Therefore, it is likely that certain cancer-derived p53 mutants transactivate growth-promoting and oncogenic genes, thereby leading to the progression of the aggressive cancers (Figure 1).

Since the mutation search for *p53* focused on the genomic region encoding the DNA-binding domain, there could still be unidentified loss of function mutations outside the DNA-binding domain [4]. Lomax *et al.* found point mutations (L344P and R337C) within the COOH-terminal oligomerization domain of p53 [47,48]. According to their results, p53 bearing L344P mutation existed as the monomeric form and lacked the transactivation ability. On the other hand, p53 carrying R377C mutation formed the tetramer, whereas this mutant displayed the significantly reduced transcriptional and pro-apoptotic activities. DiGiammarino *et al.* reported the presence of a point mutation (R337H) within the COOH-terminal oligomerization domain [49]. p53 bearing R337H mutation formed the native-like tetramer, however, its stability was significantly lower than that of wild-type p53. We have found p53ΔC lacking a part of the oligomerization domain and nuclear localization signals in human neuroblastoma-derived cell lines [50]. Based on our results, p53ΔC largely expressed in cytoplasm and had significantly lower pro-apoptotic ability as compared with wild-type p53. Therefore, p53 mutations detected outside the DNA-binding domain also caused loss of function of p53. From the clinical point of view, a novel strategy to eliminate the negative effect of mutant p53 on wild-type p53 needs to be developed. Although the great majority of p53 mutants exert a dominant-negative effect on wild-type p53, some of p53 mutants such as R175P display only partial loss of their DNA-binding activity [5].

## 4. Proteolytic Degradation of p53

Under normal conditions, p53 is maintained at a quite low level through the ubiquitin/proteasome-dependent protein degradation system. RING-finger type E3 ubiquitin protein ligase MDM2 (murine double minute 2) largely catalyzes this process [6-8]. *MDM2* was identified as one of the genes amplified on the double minute chromosomes present in the spontaneously transformed murine cell lines [51]. Since NIH3T3 cells overexpressing MDM2 are tumorigenic in nude mice, MDM2 possesses a transforming potential [52]. MDM2 binds to the NH_2_-terminal transactivation domain of p53 and catalyzes the addition of ubiquitin to a cluster of six COOH-terminal Lys residues (Lys-370, Lys-372, Lys-373, Lys-381, Lys-382 and Lys-386) in p53 [53,54]. Polyubiquitinated p53 are detectable in cells exposed to proteasome inhibitor [55]. Mutant p53 escapes from MDM2-mediated proteasomal degradation and accumulates to high levels in cancer cells [46]. Recently, it has been described that MDM2 has a post-ubiquitination function for p53 degradation [56]. According to their results, MDM2 enhanced the association of p53 with proteasome. Lai *et al.* found that MDM2 monoubiquitinates p53 but not polyubiquitinates p53 [54], suggesting that there could exist an E4 ubiquitin protein ligase which catalyzes the polyubiquitination of p53. Grossmaan *et al.* reported that p300 with an intrinsic histone acetyltransferase activity acts as an E4 ubiquitin protein ligase for p53 [57,58].

Since p53 transactivates *MDM2*, p53 creates the negative auto-regulatory feedback loop in which p53 induces the expression of its negative regulator MDM2. In addition to MDM2, RING-finger type E3 ubiquitin protein ligases Pirh2 (p53-induced RING H2 domain protein) [59] and COP1 (constitutive photomorphogenic 1) [60,61] also interact with p53 and mediate the ubiquitin/proteasome-dependent degradation of p53 in an MDM2-independent manner. Like MDM2, Pirh2 and COP1 inhibit transcriptional as well as pro-apoptotic function of p53. Since Pirh2 and COP1 are p53-induced target gene products, they also participate in a negative auto-regulatory feedback loop which controls p53. Expression studies demonstrated that COP1 is overexpressed in breast and ovarian adenocarcinomas in association with a remarkable reduction of steady-state p53 [61]. Therefore, it is likely that the overexpression of COP1 contributes to the rapid degradation of p53 in cancers and attenuates the function of p53. Alternatively, p53-interacting protein termed HAUSP (herpes virus-associated ubiquitin-specific protease) has an intrinsic enzymatic activity to deubiquitinate p53 and thereby increasing its stability even in the presence of excessive amounts of MDM2 [62].

## 5. Subcellular Distribution of p53

Appropriate nuclear distribution of p53 is critical for the expression of the transcriptional activity mediated by p53. In addition to the mutational inactivation of p53, the abnormal cytoplasmic retention of p53 causes the loss of function of p53. In contrast to the majority of other cancers, *p53* is infrequently mutated in human neuroblastoma [17]. Previously, Moll *et al.* found that wild-type p53 is largely expressed in cytoplasm in the undifferentiated neuroblastoma [63], indicating that the inability of nuclear translocation of p53 attenuates its tumor suppressive activity. Abnormal cytoplasmic localization of p53 is also observed in human primary breast cancers, colon cancers and hepatoblastomas [64,65]. Subsequent studies demonstrated that the COOH-terminal region of p53 containing NLS is masked in neuroblastoma and the addition of the short COOH-terminal peptide promotes the nuclear access of p53 [64]. Intriguingly, it has been shown that the aberrant hyperubiquitination of p53 contributes to its cytoplasmic retention in neuroblastoma [66].

Nikolaev *et al.* discovered a large cytoplasmic protein termed Parc (p53-associated, Parkin-like cytoplasmic protein) [67]. According to their results, NH_2_-terminal region of Parc interacted with the COOH-terminal region of p53. Parc had an intrinsic E3 ubiquitin protein ligase activity, however, Parc had a negligible effect on the steady-state expression level of p53. Of note, Parc was associated with the majority of cytoplasmic p53 and acted as a cytoplasmic anchor protein for p53. siRNA-mediated knockdown of Parc in neuroblastoma resulted in an increase in a chemo-sensitivity in a p53-dependent manner.

Mihara *et al.* found that, in response to DNA damage, a fraction of p53 is translocated from cell nucleus into mitochondria in cancer cells undergoing apoptosis [68]. Within mitochondria, p53 directly promotes the permeabilization of the outer mitochondrial membrane by forming complexes with BclXL and Bcl2, and thereby releasing cytocrome c into cytoplasm. Additional studies demonstrated that MDM2-mediated monoubiquitination of p53 promotes its mitochondrial translocation where p53 undergoes a rapid deubiquitination by mitochondrial HAUSP, generating the apoptotically active non-ubiquitinated p53 [69]. Down-regulation of HAUSP, results in an increased resistance to DNA damage-induced apoptosis in association with the suppression of mitochondrial translocation of p53 [70]. Thus, targeting p53 to mitochondria, which causes the dysfunction of mitochondria, might be one of the transcription-independent pro-apoptotic pathways mediated by p53.

## 6. p53-Responsible Target Gene Products

Since p53 exerts its biological function through its target gene products, it is quite important to identify p53-target gene products and also to elucidate the functional significance of them. In this section, we describe the functional significance of the representative p53-target gene products.

The cell cycle inhibitor p21 has been discovered by four independent research groups. El-Deiry *et al.* identified *p21^WAF1^* (wild-type p53-activated fragment 1) as a p53-target gene [71]. *p21^WAF1^* gene promoter contains two p53-responsive elements and its gene product suppresses cell growth. Harper *et al.* discovered p21^CIP1^ (Cdk-interacting protein) as a Cdk2 (Cyclin-dependent kinase 2)-binding protein [72]. p21^CIP1^ tightly binds to Cdk2 and inhibits its protein kinase activity to block the phosphorylation of tumor suppressor pRB (retinoblastoma susceptibility gene product). Xiong *et al.* compared the subunit composition of Cdk/cyclin complexes between normal and transformed cells, and found that p21 is frequently lost from multiprotein complex derived from transformed cells [73]. Alternatively, Noda *et al.* identified p21^SD1^ by using expression screening from senescent human diploid fibroblasts [74]. p21^SD1^ (senescent cell-derived inhibitor) blocks DNA synthesis and maintains the senescent phenotype. Now, we call it p21^WAF1^. In response to cellular stresses, p53 induces G1 cell cycle arrest through the up-regulation of p21^WAF1^.

Hermeking *et al.* described that a negative cell cycle regulator 14-3-3σ is induced in response to DNA damage in a p53-dependent manner and involved in the induction of G2/M arrest [75]. Further studies demonstrated that 14-3-3σ antagonizes Mdm2-mediated p53 degradation and p53 nuclear export [76]. Ohki *et al.* discovered a novel p53-target gene product termed Reprimo [77]. Reprimo is a highly glycosylated cytoplasmic protein which induces G2 arrest. Similarly, Tanaka *et al.* isolated p53-inducible gene product termed p53R2 with a significant sequence similarity to the ribonucleotide reductase small subunit (R2) [78]. Subsequent studies demonstrated that p53R2 is induced in response to a variety of DNA damage and causes G2/M arrest.

p53 is tightly maintained at extremely low level under normal conditions. MDM2 targets p53 for ubiquitin/proteasome-mediated degradation [6-8]. Barak *et al.* reported that *MDM2* is one of the transcriptional target genes of p53 [79]. Thus, MDM2 participates in a negative auto-regulatory feedback loop, which controls p53 expression level.

Since p53-dependent apoptosis is mediated by mitochondrial dysfunction, p53-inducible mitochondria proteins are of particular interest. We describe here BAX (Bcl2-associated X protein), p53AIP1 (p53-regulated apoptosis-inducing protein 1), NOXA (Latin for damage) and PUMA (p53 upregulated modulator of apoptosis). Selvakumaran *et al.* found that *BAX* (Bcl2-associated X protein) is an immediate early p53-responsive gene [80]. BAX is normally distributed in cytoplasm or loosely associated with mitochondrial membrane. Upon stimulation of apoptosis, BAX is induced to undergo conformational change followed by formation of membrane-inserted homo-oligomers that results in the outer mitochondrial membrane permeabilization and the releases of cytochrome c from the mitochondrial intermembrane space to cytosol [81]. Oda *et al.* identified p53AIP1 as one of the p53-target gene products [82]. p53AIP1 which is localized within mitochondria, promotes apoptosis through affecting the mitochondrial membrane potential, and thereby releasing cytochrome c [83]. p53-dependent up-regulation of p53AIP1 is tightly associated with p53 phosphorylatyion at Ser-46. NOXA has been rediscovered in a differential display approach [84]. NOXA is induced in response to DNA damage in a p53-dependent manner. It has been shown that two domains (BH3 domain and mitochondrial targeting domain) in Noxa are essential for the release of cytochrome c from mitochondria [85]. PUMA has been identified as one of p53-target gene products [86,87]. PUMA is detectable exclusively in mitochondria and is associated with pro-survival Bcl2 through its BH3 domain. PUMA induces apoptosis through the dysfunction of mitochondria. Jeffers *et al.* found that BAX is required for PUMA-mediated apoptosis, placing BAX downstream of PUMA [86].

## 7. Post-translational Modifications

Upon DNA damage, p53 is induced at protein level and exerts its transcriptional as well as pro-apoptotic function in association with chemical modifications such as phosphorylation and acetylation [2-5]. p53 is phosphorylated at Ser-15, Ser-20 and Ser-46 in response to DNA damage. NH_2_-terminal phosphorylation of p53 promotes the dissociation of MDM2 from p53/MDM2 complex, and thereby converts p53 from a latent to an active form [89]. In contrast, protein phosphatases PP-1 and PP2A dephosphorylate p53, and negatively modulates its activity [90,91]. Therefore, DNA damage-induced phosphorylation event plays a critical role in the regulation of p53 stability and also activity. Ser-15 is phosphorylated by ATM (ataxia-telangiectasia mutated) [92], ATR (ataxia-telangiectasia mutated and Rad3-related) [93], Chk1 (checkpoint kinase 1) [94], and DNA-PK (DNA-dependent protein kinase) [95]. Ser-20 is phosphorylated by Chk2 (checkpoint kinase 2) [96] and Plk3 (polo-like kinase 3) [97]. HIPK2 (homeodomain interacting protein kinase 2) and PKCdelta (protein kinase C delta) have been shown to be involved in phosphorylation of p53 at Ser-46 [98,99]. Among them, phosphorylation of p53 at Ser-46 is closely associated with its pro-apoptotic function.

In addition to NH_2_-terminal phosphorylation of p53, p53 is also phosphorylated within its COOH-terminal region. CKII (casein kinase II) phosphorylates p53 at Ser-392 [100] and PKC phosporylates p53 at Ser-371, Ser-376 and Ser-378 [101]. These COOH-terminal phosphorylations of p53 enhance its DNA-binding activity [2-4]. Under normal conditions, the COOH-terminal region of p53 masks its DNA-binding domain in a latent conformation, and acts as a negative regulator [102,103]. Because the treatment of anti-p53 antibody recognized the COOH-terminal portion of p53 or COOH-terminal truncation of p53 results in a significant increase in its DNA-binding activity [102]. In contrast, we have found that mitotic regulator Plk1 (polo-like kinase 1) inhibits transcriptional and pro-apoptotic activities of p53 through physical interaction and phosphorylation [104]. Thus, phosphorylation of p53 does not always act as an activation signal.

p53 is also acetylated in response to DNA damage [2-4]. DNA damage-induced acetylation is mediated by p300 which acts as a ubiquitous transcriptional co-activator [105]. p300 interacts with NH_2_-terminal region of p53 and mediates acetylation of a cluster of COOH-terminal Lys residues (Lys-370, Lys-372, Lys-373, Lys-392 and Lys-381) [5]. Sakaguchi *et al.* demonstrated that PCAF (p300/CBP-associated factor), another histone acetyltransferese, acetylates Lys-320 of p53 in response to DNA damage [106]. Of note, these COOH-terminal Lys residues are also the target sites for ubiquitin ligation. Thus, acetylation of p53 reduces its ubiquitination levels by competition between acetylation and ubiquitination. Indeed, p300-mediated acetylation of p53 leads to an increase in its stability and enhancement of its transcriptional as well as pro-apoptotic activity [107]. In accordance with this notion, Michishita *et al.* described that SIRT1 (silent mating type information regulation 2 homolog 1) which has an intrinsic deacetylase activity, interacts with p53 and attenuates p53-dependent cell cycle arrest, as well as apoptosis in response to DNA damage through deacetylation of Lys-382 [108]. Kawai *et al.* reported that p300 has a dual role in the regulation of p53 stability [109]. Based on their results, p300 stabilized p53 in the presence of lower level of MDM2, whereas p300 promoted MDM2-dependent proteasomal degradation of p53 in the presence of a higher level of MDM2. This might be due to the E4 ubiquitin protein ligase activity of p300 [57]. Further experiments should be conducted to address this issue.

## 8. Protein-protein Interaction

Non-catalytic protein-protein interaction is one of the regulatory mechanisms of p53 function. In this section, we describe the representative p53-binding partners which regulate p53 function.

Among 14-3-3 family members, 14-3-3σ acts as a positive regulator for p53 [76]. The expression of 14-3-3σ is undetectable in human breast cancer, gastric cancer and hepatocellular carcinoma [110-112], suggesting that loss of 14-3-3σ is crucial in the development of cancer. p53 interacts with the COOH-terminal region of 14-3-3σ (amino acid residues 153 to 248) and this interaction has a positive impact on the stability of p53 by blocking MDM2. In addition, 14-3-3σ facilitates p53 tetramer formation and enhances its transcriptional activity [76].

ASPP1 and ASPP2 which contain ankylin repeats, SH3 domain and Pro-rich domain, interact with p53 and specifically enhance pro-apoptotic activity of p53 through the inhibition of pro-survival Bcl2 [113]. Down-regulation of ASPP1 or ASPP2 results in a decrease in a sensitivity to CDDP. As expected, ASPP1 and ASPP2 increase the recruitment of p53 onto *BAX* promoter and enhance the transcriptional activity of p53. From a functional point of view, ASPP1 and ASPP2 significantly enhance p53-mediated transactivation of pro-apoptotic target genes such as *BAX* and *PIG-3* (p53-inducible gene 3), whereas ASPP1 and ASPP2 enhance p53-dependent transactivation of p53-target genes implicated in cell cycle arrest to a lesser degree. Expression levels of ASPP1 and ASPP2 is significantly reduced in human breast cancers as compared with those of corresponding normal tissues. Thus, the disregulation of ASPP1 and ASPP2 contributes to the development of breast cancer.

Iwabuchi *et al.* identified 53BP1 as a novel p53-binding partner [114]. 53BP1 binds to the central region of p53 (amino acid residues 80-320). In contrast to wild-type p53, 53BP1 does not interact with cancer-derived p53 mutants (R175H and R273H). Subsequent studies revealed that 53BP1 enhances p53-mediated transcriptional activation [115]. Rappoid *et al.* found that 53BP1 is rapidly phosphorylated by ATM in response to DNA damage, and forms discrete nuclear foci containing phosphorylated histone variant H2AX (γH2AX). γH2AX localizes at the sites of DNA strand breaks. Therefore, 53BP1 is closely involved in the early DNA damage-signaling pathway [116].

We have previously identified NFBD1 (nuclear factor with BRCT domain 1)/MDC1 (mediator of DNA damage checkpoint protein 1) with an anti-apoptotic function [117]. Further studies demonstrated that NFBD1 interacts with γH2AX through its COOH-terminal BRCT domains and recruits MRN (MRE11, Rad50 and NBS1) complex onto the sites of DNA damage to facilitate efficient DNA repair [118-120]. Like 53BP1, NFBD1 also participates in the regulation of DNA damage response. Indeed, *NFBD1*-deficient mice display chromosome instability, DNA repair defects and radiation sensitivity [121]. Recently, we have found that p53 interacts with NFBD1 [122]. According to our results, NFBD1 bound to NH_2_-terminal region of p53 and inhibited ATM-mediated phosphorylation of p53 at Ser-15 to protect cells from apoptosis during the early phase of DNA damage response. During the late phase of DNA damage response, p53 dissociated from p53/NFBD1 complex and was phosphorylated at Ser-15 to exert its pro-apoptotic function.

**Figure 2.**
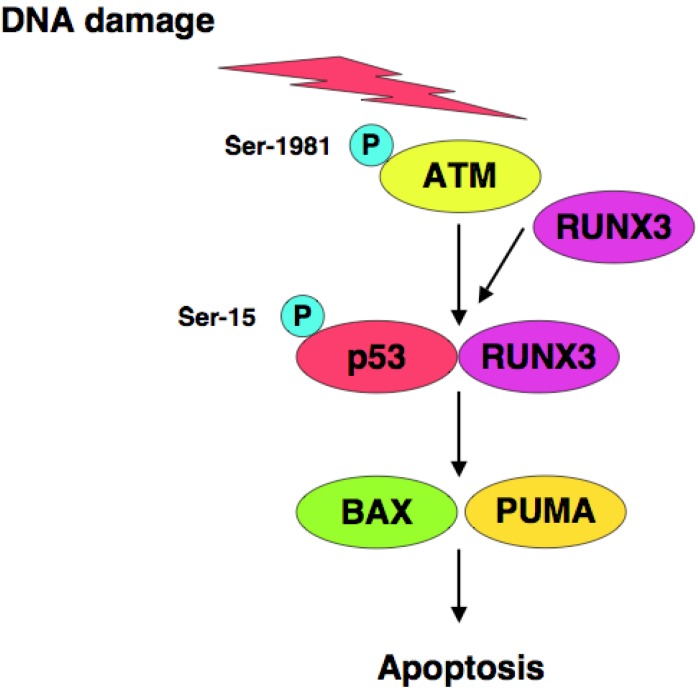
Functional interaction between p53 and RUNX3. Upon DNA damage, RUNX3 is associated with phosphorylated form of ATM (p-ATM) and recruits it to p53 to facilitate phosphorylation of p53 at Ser-15.

*RUNX3* is one of Runt-related (*RUNX*) gene family members and has been considered to be a candidate tumor suppressor for human gastric cancer [123,124], however, it remains unclear how RUNX3 exerts its tumor suppressor function. We have found that RUNX3 interacts with p53 and enhances its transcriptional, as well as its pro-apoptotic, activity [125]. Based on our results, siRNA-mediated knockdown of RUNX3 resulted in a significant decrease in sensitivity to anti-cancer drug in *p53*-proficient cells but not in *p53*-deficient cells, suggesting that there could exist a functional interaction between RUNX3 and p53 in response to DNA damage. Of note, knockdown of RUNX3 attenuated ATM-dependent phosphorylation of p53 at Ser-15 in response to DNA damage. Thus, RUNX3 recruits phosphorylated forms of ATM to p53, and thereby activates p53 (Figure 2).

## 9. Variant Forms of p53

It has long been believed that human *p53* encodes a single protein of 53 kDa. Yin *et al.* noticed the presence of the relatively smaller protein (47 kDa) which was detectable by the monoclonal anti-p53 antibody termed PAb 421 (epitope: amino acid residues 372 and 382) [126]. Further studies demonstrated that this 47 kDa protein is recognized by the monoclonal anti-p53 antibody 1801 (epitope: amino-acid residues 46 and 55), but not by the monoclonal anti-p53 antibodies DO-1 (epitope: amino acid residues 20-25) and DO-13 (epitope: amino acid residues 26-35). From close inspection of the NH_2_-terminal amino acid residues of p53, they found the second translation initiation site at Met-40. Thus, they concluded that this 47 kDa protein is generated by the alternative translational initiation, and termed p53/47. Because p53/47 lacks an NH_2_-terminal MDM2-binding site, it is not targeted for proteasomal degradation-mediated by MDM2. Additionally, p53/47 has an ability to form the homo-tetramer and hetero-tetramer with full-length p53. As mentioned above, NH_2_-terminal transactivation domain of p53 is divided into two sub-domains, TA 1 and TA II. p53/47 lacks TA I domain but retains TA II domain. Subsequent studies revealed that p53/47 is not able to transactivate *p21^WAF1^*, whereas it can preferentially increase the expression of *MDM2, GADD45* and *BAX*. These observations suggest that TA I and TA II domains differentially transactivate p53-target genes.

**Figure 3.**
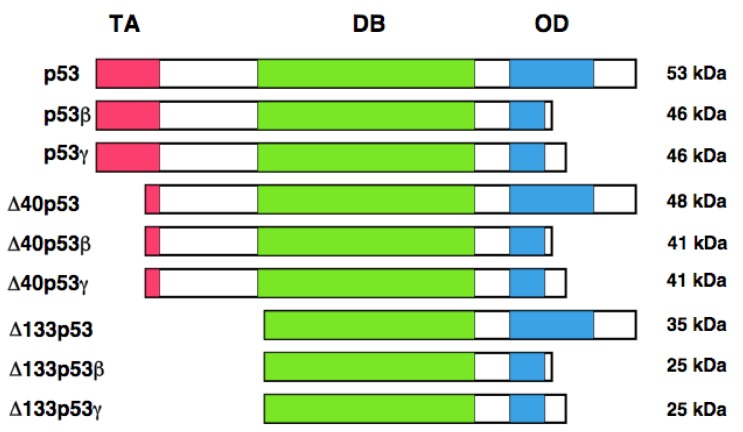
Structure of p53 variants. TA, transactivation domain; DB, sequence-specific DNA-binding domain; OD, oligomerization domain. Estimated molecular weights are also shown.

In addition to the alternative translation product of p53, Bourdon *et al.* described that human *p53* encodes multiple variants arising from alternative splicing and alternative promoter usage [127, 128]. According to their results, they found an internal promoter within intron 4 of human *p53* by using GeneRacer PCR-based strategy. Finally, they discovered the NH_2_-terminally truncated variant initiated at codon 133 (Δ133p53) distinct from p53/47. Δ133p53 lacks NH_2_-terminal transactivation domain and Pro-rich domain. They extended their study to identify the other variant forms of p53 by RT-PCR. They found that the alternative splicing event of intron 9 leads to the generation of p53β and p53γ, which delete the COOH-terminal oligomerization domain. Collectively, human *p53* encodes p53, p53β, p53γ, Δ133p53, Δ133p53β, Δ133p53γ, Δ40p53, Δ40p53β, and Δ40p53γ. Δ40p53 corresponds to p53/47 (Figure 3). Based on their results, p53β was localized largely in cell nucleus, whereas p53γ was detectable both in cell nucleus and cytoplasm. Additionally, Δ133p53β was expressed both in cell nucleus and cytoplasm; however, Δ133p53γ was detected only in cytoplasm. Extensive expression studies demonstrated that p53 variants are expressed in a wide variety of human normal tissues but in a tissue-dependent manner. In addition, DNA damage-mediated accumulation of p53β and Δ133p53β were not detectable. Intriguingly, Δ133p53 was detected in 24 out of 30 primary breast cancers. Since Δ133p53 acts as a dominant-negative inhibitor toward wild-type p53, it is possible that Δ133p53 is involved in the development of breast cancers bearing wild-type p53.

## 10. p53-mediated Therapy

The therapeutic efficiency of anti-cancer agents depends strongly on their ability to trigger apoptosis in target cancer cells [129]. Since p53 plays a pivotal role in the regulation of cell fate in response to DNA damage, the therapeutic strategies which activate p53-mediated pro-apoptotic pathway and/or eliminate the dominant-negative effect of mutant p53 on wild-type p53 should be required.

As mentioned above, MDM2 binds to the NH_2_-terminal region of p53 and inhibits its transcriptional as well as pro-apoptotic function. MDM2 also facilitates proteasomal degradation of p53. Thus, MDM2 antagonist could activate p53 and offer a novel therapeutic approach to cancer. Vassilev *et al.* discovered the first potent and selective low molecular weight inhibitor of p53-MDM2 binding termed Nutlin [130]. According to their results, Nutlin bound to MDM2 in the p53-binding pocket and blocked the interaction between MDM2 and p53, which resulted in the stabilization of p53 and also activation of p53-mediated pro-apoptotic pathway in cancer cells bearing wild-type *p53*. In addition to Nutlin, it has been shown that M1-219, which acts as an inhibitor for MDM2, might be one of the promising agents to reactivate p53 [131]. Vitali *et al.* described that a short peptide derived from p53 COOH-terminal region containing Parc-binding domain disrupts the interaction between Parc and p53 [132]. Treatment of this peptide caused the nuclear relocation of p53 and increased in sensitivity to anti-cancer drug in cancers such as neuroblastoma with wild-type cytoplasmic p53.

Alternatively, the re-activation of mutant p53 contributes to much more efficient treatment of cancers bearing mutant *p53*. After screening of a library of low-molecular-weight compounds, Bykov *et al.* found that one compound termed PRIMA-1 has an ability to restore wild-type function to mutant p53 such as R248Q and R175H [133]. Further studies demonstrated that PRIMA-1 binds to the DNA-binding domain of mutant p53 and covalently modifies the thiol groups in the central core DNA-binding domain, and thereby reactivates mutant p53 [134].

## 11. Conclusions

Since over 50% of human cancers carry p53 mutations, mutational inactivation is a major molecular mechanism behind p53 dysfunction. Cancers bearing p53 mutation sometimes display a chemo-resistant phenotype. Although the intracellular balance between the expression levels of wild-type p53 and mutant p53 might be a critical determinant of cell fate in response to DNA damage, mutant p53 acts as a dominant-negative inhibitor toward wild-type p53 and exhibits a longer half-life than wild-type p53. Thus, the development of novel strategies to re-activate mutant p53 is required to provide clues to effectively treat malignant cancers bearing *p53* mutations.
